# Supplementary material for: Structural basis for the adsorption of a single-stranded RNA bacteriophage
Source: Nat Commun. 2019 Jul 16;10:3130. doi: 10.1038/s41467-019-11126-8 (PMC6635492; doi:10.1038/s41467-019-11126-8)
Supplement: Supplementary file 1 — Supplementary information [file 41467_2019_11126_MOESM1_ESM.pdf]

# Supplementary information

## Structural basis for the adsorption of a single-stranded RNA bacteriophage

Meng et al.

Supplementary figures 1-7
Supplementary table 1

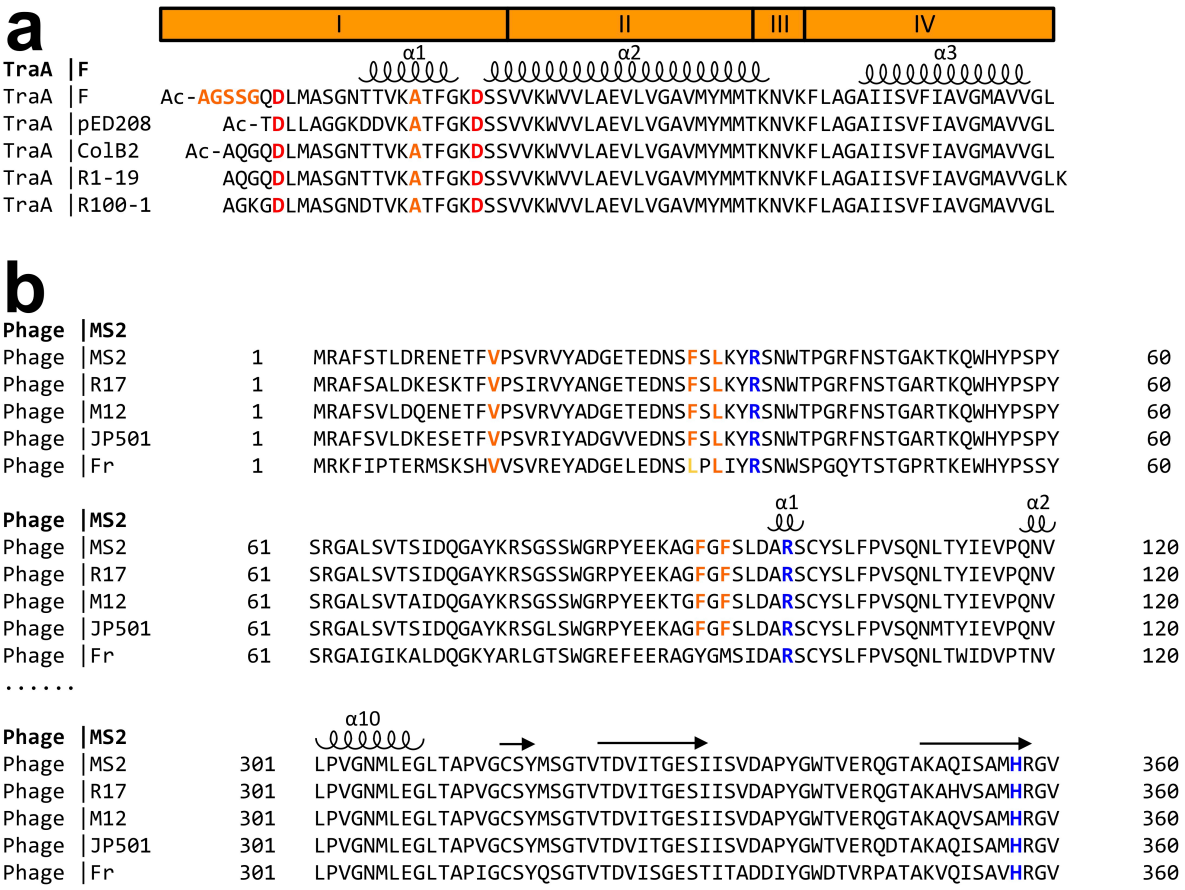


**Supplementary figure 1. The sequence alignment for the F-like pilins and the Mat of MS2-like phages.** (**a**) The sequence comparison among five F-like pilins. (**b**) The sequence comparison among the Mats of MS2 and MS2-like phages. The residues involved in the pilus/Mat interactions or indicated in previous mutagenesis studies are labeled blue (positively charged), red (negatively charged) or orange (AGSSG loop or hydrophobic). Residue 31 in the Mat of Phage Fr is a Leu instead of a Phe, but still hydrophobic, and is labeled yellow.

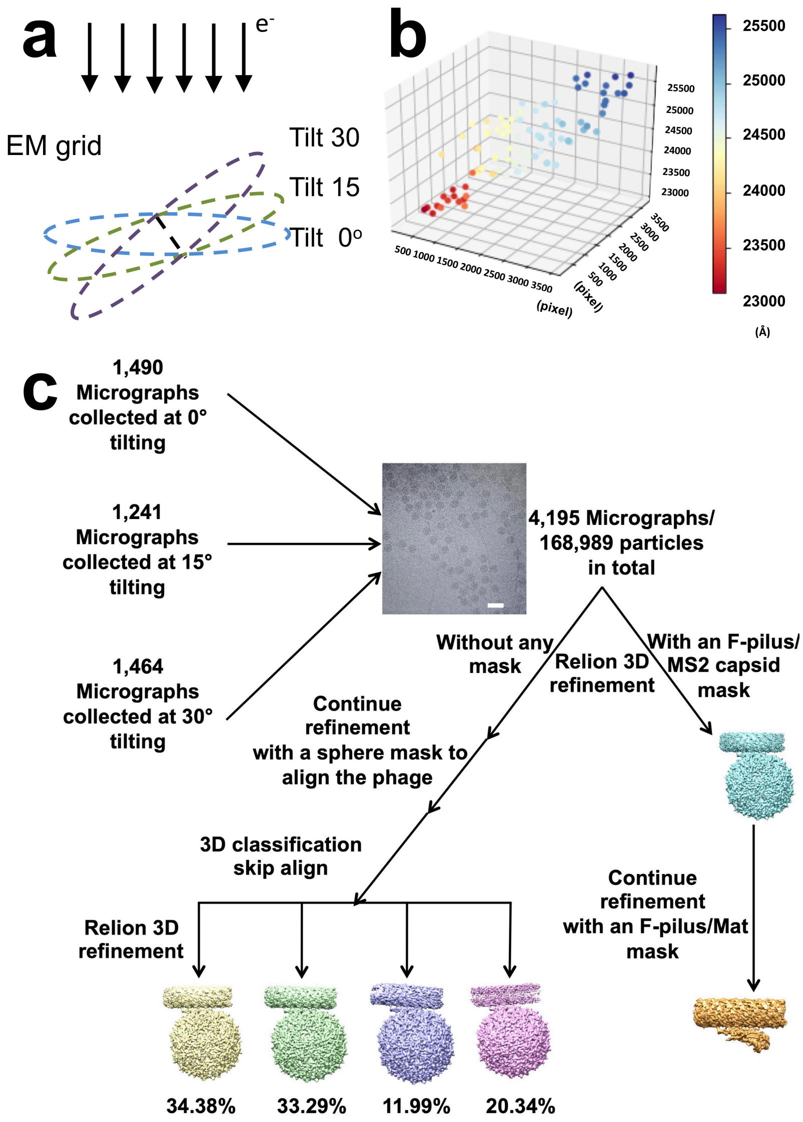


**Supplementary figure 2. The strategies for data collection and processing.** (**a**) The tilting strategy used in data collection. (**b**) The defocus values for particles in a 30°-tilted micrograph. The X-axis and Y-axis label particle coordinates in the micrograph (in pixels). The Z-axis labels the defocus values (in Å). Each dot represents one particle. The dots are colored from red to blue representing defocus values from small to large. (**c**) 1,490, 1,241, and 1,464 micrographs were collected with 0°, 15° and 30° tilting, respectively. The contrast transfer functions (CTF) were estimated by Gctf. 168,989 clean particles were used as input for the 3D auto-refine in Relion with or without a mask around the F-pilus and the MS2 capsid. To further align the local region of the F-pilus/Mat complex, the refinement was continued with a mask around the F-pilus/Mat complex. To analyze the flexibility of the MS2/F-pilus complex, 3D refinement was first continued with a spherical mask to align the phage, then 3D classification was performed with a mask around the F-pilus with the alignment skipped. Particles were separated into four classes with 34.38% in Class 1, 33.29% in Class 2, 11.99% in Class 3, and 20.34% in Class 4. The density for the F-pilus was shown as two layers in Class 4, which was due to the preferred orientations. The scale bar in the raw micrograph represents 500 Å.

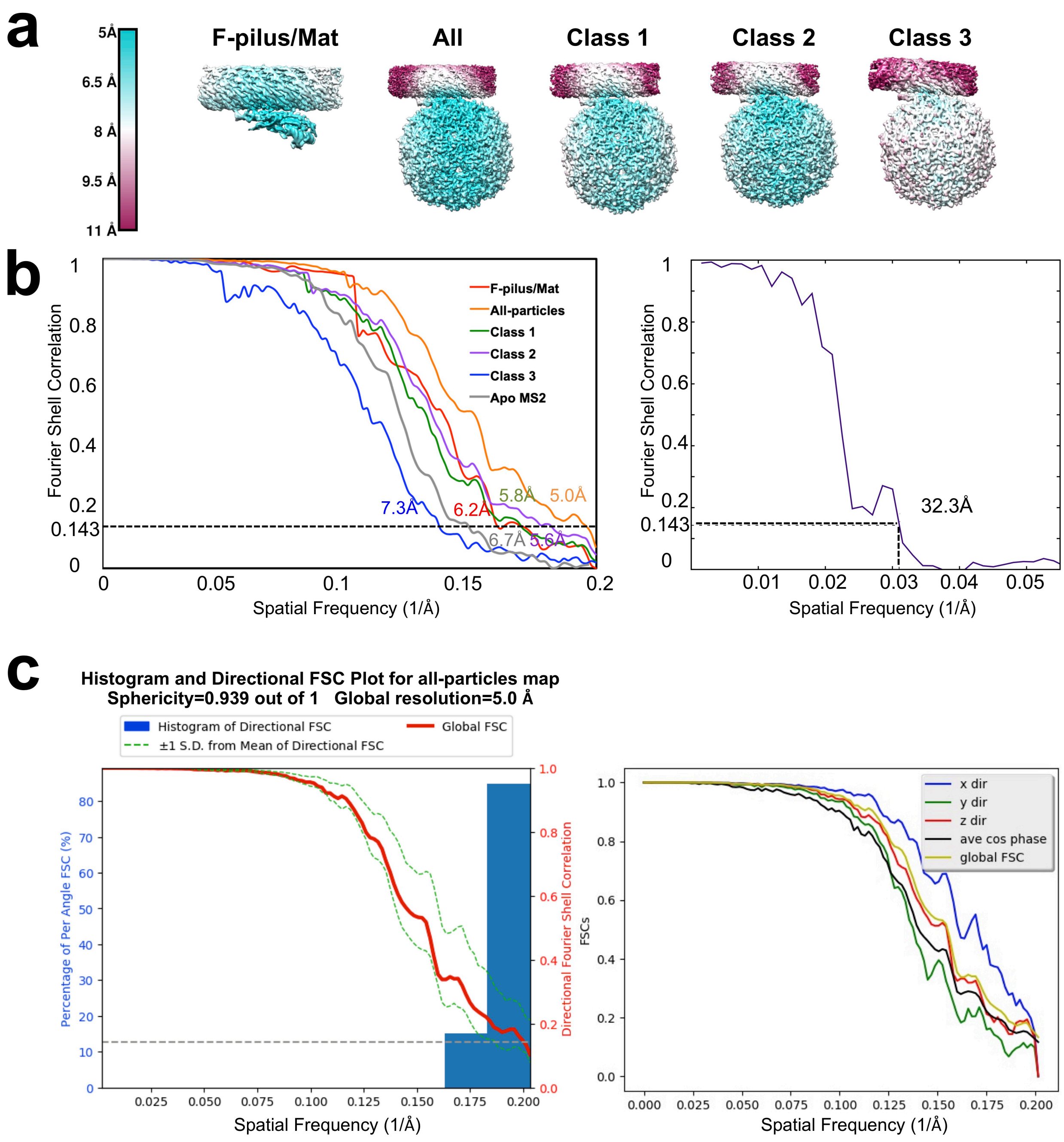


**Supplementary figure 3. Local resolutions and Fourier Shell Correlation (FSC) curves.** (**a**) Local resolutions were estimated by the Bsoft package and colored from maroon to cyan representing resolutions from low to high [1]. (**b**) (**Left**) The FSC curves for the single-particle map of the F-pilus/Mat complex, all-particles, Class 1, Class 2, Class 3, and Apo MS2 are shown in red, orange, green, purple, blue, and gray, respectively. (**Right**) The FSC curve for the MS2/F-pilus tomographic reconstruction. (**c**) Directional FSC for the MS2/F-pilus all-particles map [2].

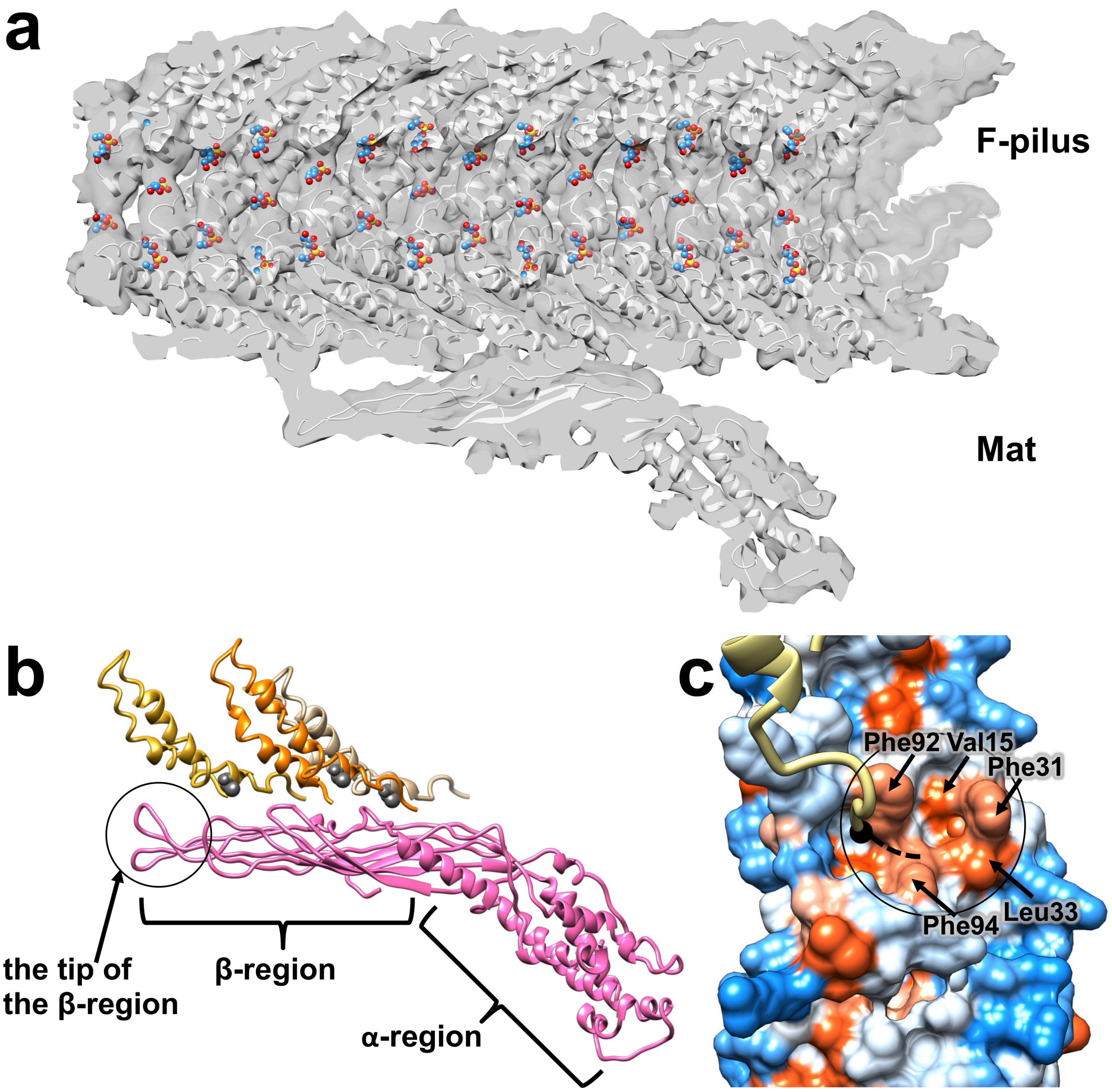


**Supplementary figure 4. The structure of the F-pilus, bound with MS2, is consistent with the previously published structure of the apo F-pilus.** (**a**) Phospholipids are visible in the map of the F-pilus/Mat complex. Pilin proteins and the Mat are colored in white. Phospholipids are colored by element. Oxygen, phosphorus, and carbon are shown in red, gold, and blue, respectively. (**b**) The Ala18 residues exposed at the pilus-Mat interface are labeled by gray spheres. The α-region, the β-region, and the tip of the β-region are labeled by brackets and a black circle, respectively. (**c**) N-terminus of one pilin (labeled by a black sphere at the end of a yellow ribbon model) extends into a hydrophobic pocket (enclosed by a black oval) consisting of Val15, Phe31, Leu33, Phe92, and Phe94 from the Mat. The dashed black line denotes the potential location of the five missing residues (AGSSG) at the N-terminus of one pilin. The surface of the Mat is colored from dodger blue for the most hydrophilic, to orange red for the most hydrophobic.

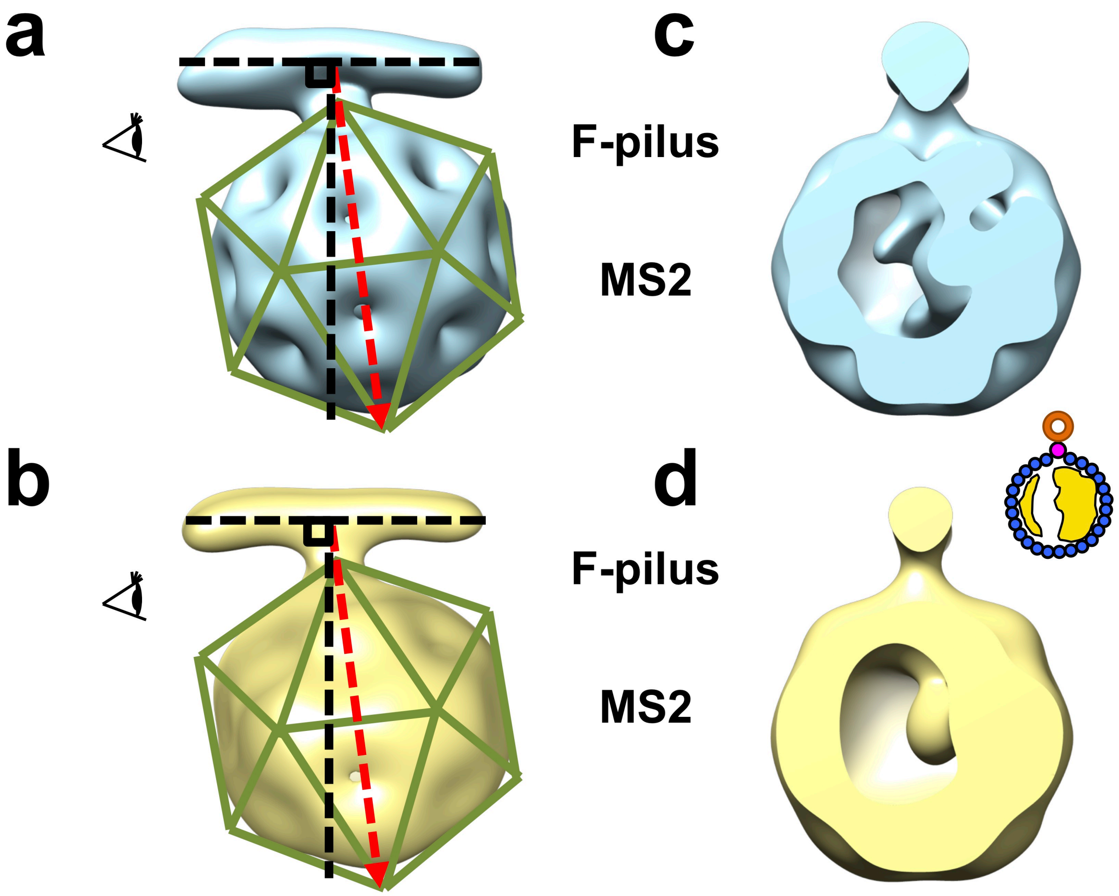


**Supplementary figure 5. The low-pass filtered single-particle cryo-EM map showing asymmetric features.** (**a, b**) Side views of the low-pass filtered single-particle cryo-EM maps of the MS2/F-pilus complex at 60-Å (panel a) and 80-Å (panel b) resolutions showing a tilting angle between the pilus axis and the two-fold symmetry axis of the capsid. The axis of the F-pilus is labeled by a horizontal dashed black line with another dashed black line perpendicular to it. The dashed red arrow indicates the two-fold axis on the side of the near-icosahedral capsid. Green lines indicate the icosahedral lattice of the capsid. (**c, d**) Cut-in views of the low-pass filtered single-particle cryo-EM map of the MS2/F-pilus complex viewed from the orientation labeled by the eye cartoons in Panels a and b, showing the unevenly distributed gRNA density. The inset shows a cartoon model for the asymmetric distribution of the gRNA.

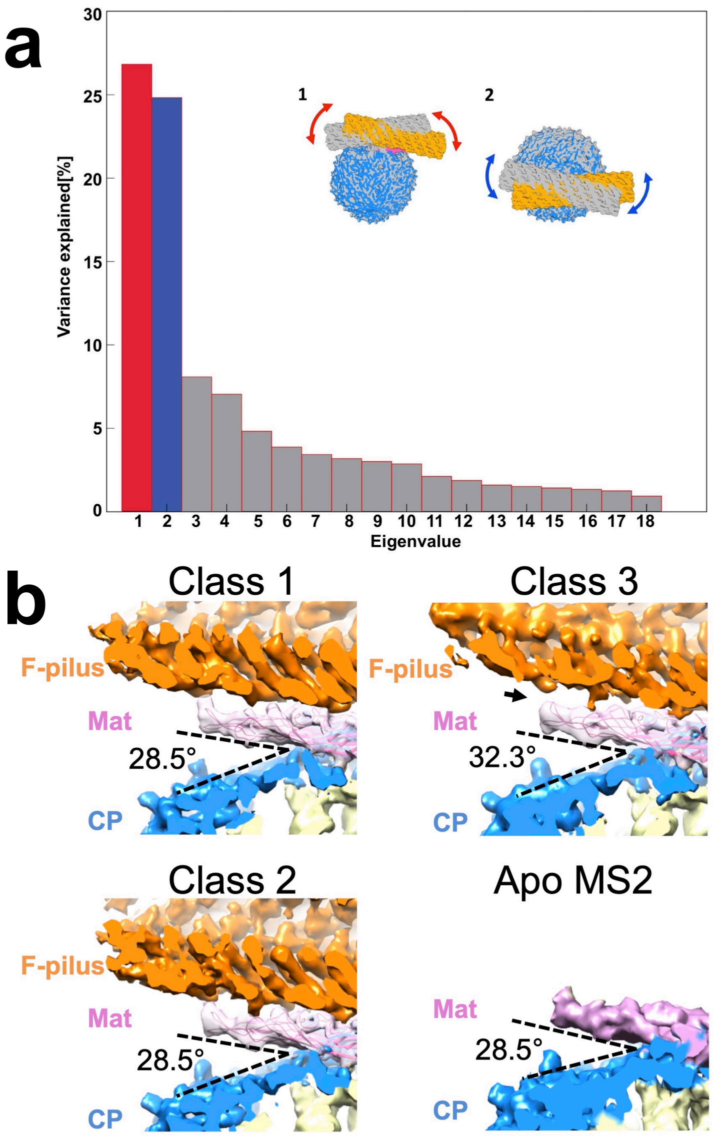


**Supplementary figure 6. Flexibility in the MS2/F-pilus complex.** (**a**) The MS2/F-pilus complex flexibility showed by the principal component analysis. Relion multibody refinement and principal component analysis were used to analyze the internal flexibility of the MS2/F-pilus complex. With the eighteen eigenvectors obtained, two principal eigenvectors can describe >50% of the variance, which represents the major flexibility in the MS2/F-pilus complex. These two eigenvectors represent mostly the tilting and the swiveling of the F-pilus against the MS2 particle, respectively. Insets show the cartoons describing the tilting (red) and swiveling (blue) motions represented by these two principal eigenvectors. (**b**) Zoom-in views of the tip of the β-region and the F-pilus. The angle between the β-region of the Mat and the phage capsid are 28.5° in Classes 1 and 2. The tip of the β-region interacts with the N-terminus of the F-pilin in Classes 1 and 2. The angle between the β-region of the Mat and the phage capsid is 32.3° in Class 3 and the interaction between the tip of the β-region and the F-pilus is disrupted. A black arrow points to the broken density. The angle between the β-region of the Mat and the phage capsid is 28.5° in the Apo state, which is the same as in Classes 1 and 2.

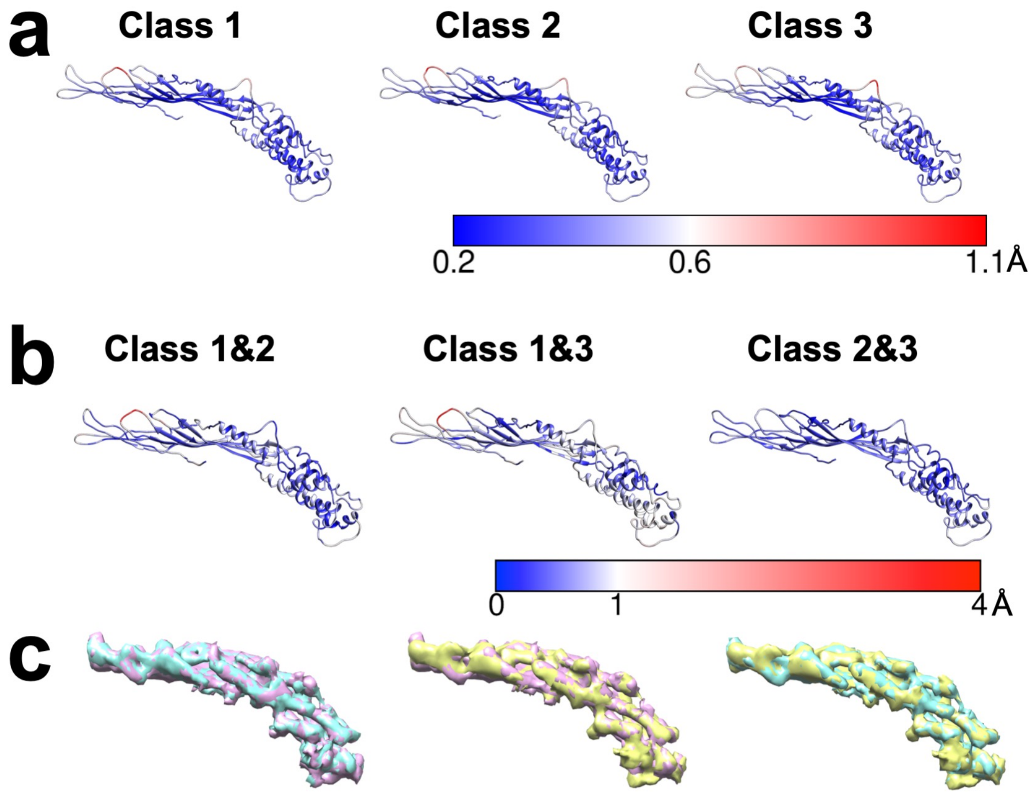


**Supplementary figure 7. Structural comparisons of the Mats from Classes 1, 2 and 3 reveal no detectable variation in the tertiary structure.** To estimate the fitting precision of MDFF, we first calculated the average and standard deviation (error) for the fitted models as follows. For each of the three conformations, we extracted 200 frames from the MDFF trajectory after the simulation converged. Each frame represents a fitted conformation, which fluctuates around an average conformation. We then estimated the mean and standard deviation (error) for the position of each Cα atoms among these 200 models for each conformation. The standard deviations of the Cα position are colored-coded on the model in Panel a and between 0.25 Å (blue) ~1.24 Å (red), which are smaller than the resolution of the map (5.6-7.3 Å). This is due to the fact that the MDFF fitting uses not only the information from the density map but also the molecular energy, which maintains a stereochemically correct and stable structure. We then estimated the difference among the three conformations by calculating the Cα deviation between each pair of the average conformations. Panel b shows the Cα deviations between each pair of conformations, color-coded on the model, which are between 0.06 Å (blue) ~ 4.63 Å (red), with the largest deviations in the flexible loop regions. Overall, the models of the Mat in the three conformations are very similar with most of the regions showing a Cα deviation of less than 1 Å. Moreover, the direct overlapping of the density maps of the three conformations, in Panel c, shows high cross correlations of 0.97 between Class1 and Class2, 0.96 between Class1 and Class3, and 0.96 between Class2 and Class3, which means the three density maps of the Mat are very similar to each other. In summary, at our current resolutions of the maps, there is no detectable variation in the tertiary structure of the Mat in these three conformations of the pilus-bound MS2.

## Supplementary table 1. Cryo-EM data collection, refinement and validation statistics

| | #1 F-pilus/Mat (EMDB-9397) (PDB 6NM5) | #2All particles (EMDB-0453) | #3 Class 1 (EMDB-9399) | #4 Class 2 (EMDB-0448) | #5 Class 3 (EMDB-0450) | #6 Apo MS2 (EMDB-0451) | #7 MS2/F- pilus (Tomographic reconstruction) (EMDB-0338) |
|---|---|---|---|---|---|---|---|
| **Data collection and processing** | | | | | | | |
| Magnification | JEM3200FSC 30,000x | JEM3200FSC 30,000x | JEM3200FSC 30,000x | JEM3200FSC 30,000x | JEM3200FSC 30,000x | FEI TF20 29,000x | Polara G2 9,400x |
| Voltage (kV) | 300kv | 300kv | 300kv | 300kv | 300kv | 200kv | 300kv |
| Electron exposure (e–/Å$^2$) | ~37 | ~37 | ~37 | ~37 | ~37 | ~34 | ~80 |
| Defocus range (μm) | -1~-3.5 | -1~-3.5 | -1~-3.5 | -1~-3.5 | -1~-3.5 | -1~-3.5 | -6~-9 |
| Pixel size (Å) | 1.23 | 1.23 | 1.23 | 1.23 | 1.23 | 1.25 | 4.45 |
| Symmetry imposed | C1 | C1 | C1 | C1 | C1 | C1 | C1 |
| Particle images (no.) | 168,989 | 168,989 | 60,004 | 58,447 | 17,467 | 44,987 | 1,245 subtomograms |
| Map resolution (Å) | 6.2 | 5.0 | 5.8 | 5.6 | 7.3 | 6.7 | 32.2 |
| FSC threshold | 0.143 | 0.143 | 0.143 | 0.143 | 0.143 | 0.143 | 0.143 |
| **Refinement** | | | | | | | |
| Initial model used (PDB code) | 5LER and 5TC1 | | | | | | |
| Model composition | | | | | | | |
| Non-hydrogen atoms | | | | | | | |
| Protein residues | 5,268 | | | | | | |
| Ligands | 70 | | | | | | |
| R.m.s. deviations | | | | | | | |
| Bond lengths (Å) | 0.0033 | | | | | | |
| Bond angles (°) | 0.93 | | | | | | |
| Validation | | | | | | | |
| MolProbity score | 1.97 | | | | | | |
| Clashscore | 5.06 | | | | | | |
| Rotamer outliers (%) | 0.00 | | | | | | |
| Ramachandran plot | | | | | | | |
| Favored (%) | 82.56 | | | | | | |
| Outliers (%) | 0.02 | | | | | | |

## Supplementary References


1. Cardone, G. J.B. Heymann, and A.C. Steven. One number does not fit all: mapping local variations in resolution in cryo-EM reconstructions. *J Struct Biol* **184**, 226-236 (2013).

2. Tan YZ*, et al.* Addressing preferred specimen orientation in single-particle cryo-EM through tilting. *Nat Methods* **14**, 793-796 (2017).
