# Supplementary material for: Structural basis for the adsorption of a single-stranded RNA bacteriophage
Source: Nat Commun. 2019 Jul 16;10:3130. doi: 10.1038/s41467-019-11126-8 (PMC6635492; doi:10.1038/s41467-019-11126-8)
Supplement: Supplementary file 3 — Description of Additional Supplementary Files [file 41467_2019_11126_MOESM3_ESM.docx]

**Description of Additional Supplementary Files**

**File Name: Supplementary Movie 1**

**Description:** The overall architecture of the MS2/F-pilus complex with the F-pilus colored orange, the Mat colored pink, coat protein blue and the gRNA yellow.

**File Name: Supplementary Movie 2**

**Description:** The relative moving direction between the F-pilus and the MS2 represented by PCA Eigenvector 1.

**File Name: Supplementary Movie 3**

**Description:** The relative moving direction between the F-pilus and the MS2 represented by PCA Eigenvector 2.
